# Theranostic Potential of Adaptive Cold Atmospheric Plasma with Temozolomide to Checkmate Glioblastoma: An In Vitro Study

**DOI:** 10.3390/cancers14133116

**Published:** 2022-06-25

**Authors:** Vikas Soni, Manish Adhikari, Li Lin, Jonathan H. Sherman, Michael Keidar

**Affiliations:** 1 Micro-Propulsion and Nanotechnology Laboratory, Department of Mechanical and Aerospace Engineering, The George Washington University, Science and Engineering Hall, 800 22nd Street, NW, Washington, DC 20052, USA; manishadhikari85@gmail.com (M.A.); lilin@gwu.edu (L.L.); 2 Department of Neurosurgery, Rockefeller Neuroscience Institute, West Virginia University, 880 N Tennessee Avenue, Suite 104, Martinsburg, WV 25401, USA; jsherman0620@gmail.com

**Keywords:** cold atmospheric plasma (CAP), reactive oxygen species, glioblastoma, temozolomide, cell death, sensitization, cancer, apoptosis, caspase-3, lactate dehydrogenase (LDH), cancer therapy, in vitro, microscopy, T98G, A172

## Abstract

**Simple Summary:**

Glioblastoma (GBM) is an aggressive form of brain cancer. Here, we present a combination therapy of cold atmospheric plasma (CAP) and temozolomide (TMZ) to treat GBM in vitro. We analyze the effects of the co-treatment in two GBM (TMZ-resistant and -sensitive) cell lines. The aim of this study is mainly to sensitize these cells using CAP so that they respond well to TMZ. We further found that the removal of cell culture media after CAP treatment does not affect the sensitivity of CAP to cancer cells but enhances the effects of TMZ. However, it was observed in our study that keeping the CAP-treated media for a shorter time did not significantly inhibit T98G cells. Interestingly, keeping the same plasma-treated media for a longer duration resulted in a decrease in cell viability. On the contrary, TMZ-sensitive cell A172 responded well to the co-treatment. This could be a potential reason for the sensitization of the combination therapy.

**Abstract:**

Cold atmospheric plasma (CAP) has been used for the treatment of various cancers. The anti-cancer properties of CAP are mainly due to the reactive species generated from it. Here, we analyze the efficacy of CAP in combination with temozolomide (TMZ) in two different human glioblastoma cell lines, T98G and A172, in vitro using various conditions. We also establish an optimized dose of the co-treatment to study potential sensitization in TMZ-resistant cells. The removal of cell culture media after CAP treatment did not affect the sensitivity of CAP to cancer cells. However, keeping the CAP-treated media for a shorter time helped in the slight proliferation of T98G cells, while keeping the same media for longer durations resulted in a decrease in its survivability. This could be a potential reason for the sensitization of the cells in combination treatment. Co-treatment effectively increased the lactate dehydrogenase (LDH) activity, indicating cytotoxicity. Furthermore, apoptosis and caspase-3 activity also significantly increased in both cell lines, implying the anticancer nature of the combination. The microscopic analysis of the cells post-treatment indicated nuclear fragmentation, and caspase activity demonstrated apoptosis. Therefore, a combination treatment of CAP and TMZ may be a potent therapeutic modality to treat glioblastoma. This could also indicate that a pre-treatment with CAP causes the cells to be more sensitive to chemotherapy treatment.

## 1. Introduction

Cancer is one of the major foremost causes of morbidity and mortality worldwide. Cancer statistics show that the USA is more prone to brain cancer as compared to other forms of cancers [1]. Glioblastoma (GBM) is the most aggressive and malignant form of brain cancer and is well known as one of the utmost formidable forms of cancer due to the complex structure of the brain [2]. Hence, therapeutic strategies are needed for treating GBM. High chances of relapse and poor prognosis additionally lead to the high mortality rate of GBM [3]. The latest survey made by the American Cancer Society in 2020 revealed that patients with GBM showed a 75.42% mortality rate, which is high compared to other forms of cancers [4]. A market insight report notes that the global brain tumor treatment market has grown and will continue to grow at a compound annual growth rate of 11.5% from 2019 to 2025. Many international pharmaceutical manufacturers, including Bristol Myers Squibb, Genentech, Pfizer, Merck, etc., are focused on the preventive and cost-effective treatment of GBM. The strategies used to treat GBM are chemotherapy, radiation therapy, targeted therapy, immunotherapy, and surgery, none of which have led to a significant increase in overall survival. Surgery, although invasive, is currently the only known effective method to treat GBM. The major drawback and difficulty in GBM therapy is the resistance of brain cancer cells to traditionally used chemotherapeutic drugs [5]. Most GBM-targeted therapies require high doses of the drug to enter the blood–brain barrier, leading to systemic toxicity [6]. Consequently, effective non-invasive methods can lead to lower morbidity and perhaps provide a more efficient way of treating GBM [7].

The present standard treatment for GBM patients is temozolomide (TMZ), which is given orally after surgical GBM resection in combination with radiation therapy [8]. TMZ is an oral, alkylating, chemotherapeutic pro-drug that clears the blood–brain barrier [9]. TMZ induces apoptosis by adding a methyl group to the nitrogen-containing bases in the DNA and killing GBM [10]. However, nearly half of GBM patients are resistant to TMZ because of the presence of the methyl guanine methyltransferase (MGMT) DNA repair system [11]. The MGMT gene is responsible for the relocation of the methyl group from guanine, leads to the repair of damaged DNA, and inhibits TMZ’s cytotoxic effects on GBM cells [12]. Some studies also report that an initial response to TMZ often leads to acquired resistance by GBM cells, which is another major issue in treating GBM patients [13]. As the most promising option for GBM treatment known so far, there is an urgent need for a combination strategy of TMZ along with a non-invasive approach that increases TMZ efficacy and improves the clinical treatment of GBM.

Cold atmospheric plasma (CAP) could act as an enhancer of the TMZ effect on GBM [14]. It is made when a high voltage is applied between two electrodes and a feeder gas such as Helium is injected into the system [15,16]. CAP is composed of charged particles, reactive species, neutral particles, electromagnetic fields, and ultraviolet radiation. Presently, CAP has been extensively used in many fields of medicine including wound healing, sterilization, agriculture, etc. [17,18,19,20,21]. It is also reported in cancer treatment by direct or indirect methods through the bone [14,22,23,24]. CAP and its derived cell culture media successfully reduced the growth of xenograft tumors in mice through direct and indirect treatment above the skin layer [25,26]. Many factors play a role in reducing cancer cells and tumor growth during CAP treatment because it contains a cocktail of short-lived and long-lived reactive species (oxygen and nitrogen species), UV rays, electromagnetic waves, etc. [27,28]. It is well established that a small dose of CAP produces fewer RONS which assists the induction of cell proliferation, while a high CAP dose produces more RONS that can damage protein and all nucleic acids, resulting in apoptosis [29,30,31]. In previous studies, our group has suggested that CAP can selectively inhibit cancer cells without damaging normal cells when applied at suitable dosages and coined the term ‘Plasma sensitization’ [14,32,33,34,35]. Currently, adequate literature is available that specifies the use of CAP technology as a potent candidate for combination therapy with many other compounds and nanoparticles to treat different types of cancers in vitro and in vivo [36,37,38]. Since GBM is considered a highly malignant and aggressive form of brain cancer characterized by fast growth with extensive angiogenesis and resistance to all current known therapies [39], cold atmospheric plasma can be used along with TMZ to investigate the synergistic inhibition of GBM. A recent study showed that the combination of TMZ and CAP treatment results in a decrease in U-87 MG cells at 50 µM TMZ concentration and 180 s CAP treatment [40]. CAP also enhanced the combination effect of TMZ-CAP in vitro and in vivo and inhibited tumor growth by 78% in the U-87MG intracranial xenograft murine model [14].

Henceforth, the aim of this study is to develop a novel synergistic therapeutic model by combining the treatment of CAP with TMZ in two different GBM cell lines: T98G (TMZ-resistant) and A-172 (TMZ-sensitive). These cell-based studies will suggest the strong influence of CAP, leading to better GBM inhibition while using a smaller recommended TMZ dosage. Hence, the results present evidence of the plausible potential role of CAP and TMZ to inhibit the progression of GBM in future clinical trials. The data also suggest the potential sensitization of GBM using CAP.

## 2. Materials and Methods

### 2.1. Chemicals and Kits

Dulbecco’s Modified Eagle Medium (DMEM, Life Technologies, Washington, WA, USA) supplemented with 10% (*v*/*v*) fetal bovine serum (GE Healthcare, SH30396), 1% (*v*/*v*) penicillin and streptomycin (Life Technologies), trypsin-EDTA, and DAPI were purchased from Thermo Fisher Scientific, Waltham, MA, USA. 3-(4,5-dimethylthiazol-2-yl)-2,5-diphenyltetrazolium bromide (MTT), TMZ, and DMSO were purchased from Sigma Aldrich, St. Louis, MO, USA. PBS and isopropanol were obtained from VWR International, Radnor, PA, USA. LDH Cytotoxicity Colorimetric Assay Kit II and CaspGLOW™ fluorescein active caspase-3 staining kit were purchased from BioVision, Milpitas, CA, USA. Glass-bottom culture dishes were procured from Nest™, Westwood, NJ, USA. 

### 2.2. Cell Culture

Two human glioblastoma cell lines, T98G (CRL-1690™) and A172 (CRL-1620™), were used in the study. They were obtained from the American Type Culture Collection (ATCC). The cells were cultured in DMEM high-glucose cell culture media supplemented with 10% fetal bovine serum, 100 U/mL penicillin, and 100 mg/mL streptomycin (1% pen/strep) and were maintained at 37 °C in a 5% (*v*/*v*) humidified CO_2_ environment. The passage number used for the studies was 12 for T98G cells and 03 for A172 cells, respectively. For the combination methods, both cell lines were incubated at different time intervals with TMZ after CAP treatment.

### 2.3. CAP Jet Device Configuration

**Figure 1:**
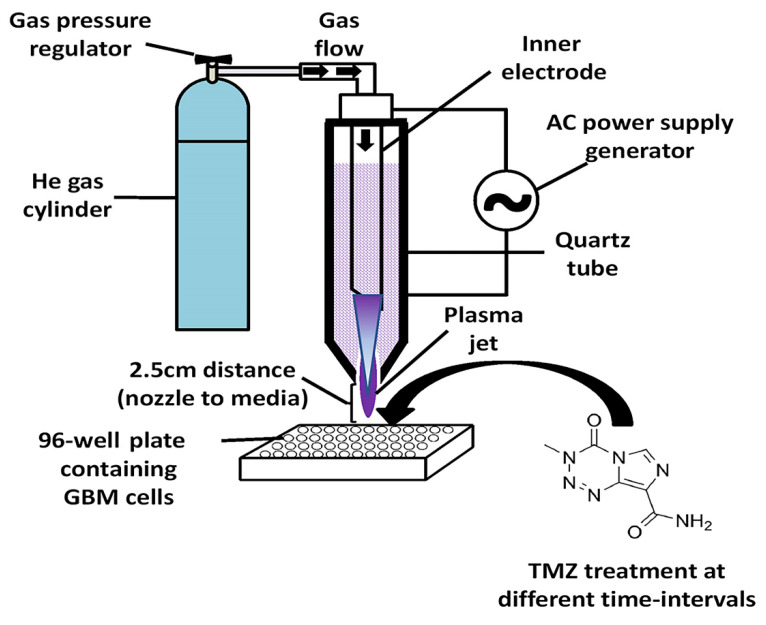
Schematic representation of the experimental setup: GBM cell treatment in a 96-well plate using the CAP jet device.

**Figure 2:**
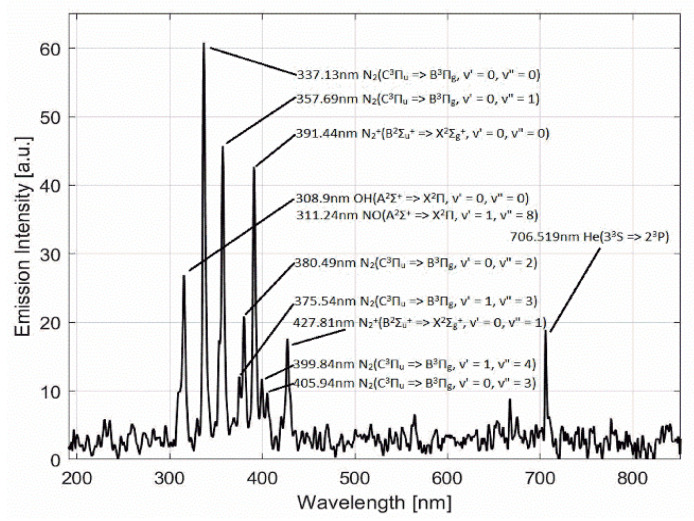
Optical emission spectrum (OES) composition of the He jet CAP source between 200 and 800 nm.

The CAP jet generator was prepared at the micro-propulsion and nano-technology laboratory at George Washington University under the supervision of Prof. Michael Keidar. The device consisted of a copper electrode wrapped around the quartz nozzle (10 mm diameter) assembled with a central electrode. The discharging frequency was 10 kHz with a helium flow rate of 4 L/min. The distance between the nozzle and the upper surface of the cell culture medium was 2.5 cm. A direct treatment method was used to treat cells in the cell culture medium, which was pretreated with a specific dose of TMZ depending on the type of GBM cell line used for the study. A schematic representation of the CAP jet and experimental setup is shown in Figure 1. The CAP jet was characterized using optical emission spectroscopy (OES) shown in Figure 2.

### 2.4. Preparation of Temozolomide (TMZ) Dilutions and IC50 of GBM Cell Lines

The drug TMZ was prepared and diluted by dissolving in DMSO and making a stock solution of 10 mg/mL. The percentage of DMSO while making TMZ stock solutions was maintained under 1%. All the working concentrations were prepared by serial dilutions from the TMZ stock in DMEM media. TMZ-resistant T98G and TMZ-sensitive A172 cell lines were seeded (in biological triplicates) in 96-well flat-bottom plates (SKU: TC10-096, Stellar Scientific, Owings Mills, MD, USA). The cells were seeded at a cell density of 5 × 10^3^ cells per well. The cells were incubated overnight in a CO_2_ incubator at 37 °C. The next day, the cells were incubated with different concentrations of TMZ from Day 1 to Day 5, respectively. The cell viability was measured by a 3-(4,5-dimethylthiazol-2-yl)-2,5-diphenyltetrazolium bromide (MTT) assay. In this, 100 μL of MTT solution (0.7 mg/mL) was added to each test well plate followed by 3 h incubation. Afterward, the incubated solution was discarded and replaced with 100 µL of MTT solvent (0.4% (*v*/*v*) HCl in anhydrous isopropanol). MTT was reduced, giving purple formazan crystal from the live cells. After these, purple-colored formazan crystals were dissolved, their absorbance was measured at 570 nm with a microplate reader (Synergy H1 Hybrid).

### 2.5. Time-Dependent CAP Sensitization of GBM Cell Lines

In this experiment, GBM cell lines T98G and A172 were seeded in 96-well plates (in biological triplicates) and divided into two groups. In the first group, media were removed directly after giving CAP treatment or TMZ in the mentioned time intervals (15 s, 30 s, 60 s, 90 s, and 120 s). In the second group, the cells remained in the CAP-treated media and TMZ for up to 72 h to study the CAP sensitization process. The dose of TMZ was maintained at ~400 μM (IC50 on Day 4) for T98G cells and ~200 μM (IC50 on Day 3) for A172 cells, respectively, from the previous results. Furthermore, experiments were conducted using different conditions in which CAP treatment time, incubation with CAP, and the number of CAP treatment times performed were varied. In all experiments, the absorbance was recorded after 72 h incubation, and absorbance was measured using the MTT assay as described above.

### 2.6. CAP-Induced TMZ-Supported Apoptosis Detection in GBM Cells by DAPI Staining

Both GBM cell lines (T98G and A172) were seeded (in biological triplicates) and cultured in DMEM media overnight in a glass-bottom culture dish of 35 mm^2^ (Nest™, Westwood, NJ, USA) before treatment with a CAP jet using, He feeder gas. The cells were divided into four groups: control, CAP, TMZ, and co-treatment (CAP + TMZ). Control cells were untreated and the CAP-treated group received a 60 s treatment, while the TMZ group received 400 μM (IC50 on Day 4) for T98G cells and 200 μM (IC50 on Day 3) TMZ for A172, respectively. In the co-treatment group, CAP was applied after the desired concentration of TMZ application to the specific cell lines. Afterwards, cells were incubated for 72 h in a CO_2_ incubator. After incubation, the cells were fixed with 3.7% paraformaldehyde and washed 2X with sterile PBS. Subsequently, the cells were permeabilized with 0.5% Triton X-100 in PBS for 5 min and stained with DAPI (1 mg/mL) for 30 min at room temperature. The cells were again re-washed with PBS to avoid excessive staining, and fluorescent images were captured using a Zeiss spinning-disk confocal microscope (Oberkochen, Germany).

### 2.7. Estimation of Lactate Dehydrogenase (LDH) and Caspase-3 Activity in GBM Cells

LDH is a cytosolic enzyme known as an indicator of cellular cytotoxicity. The LDH enzyme quantification was performed in GBM cell lines (T98G and A172). LDH is released in the cell media when the cell is damaged due to CAP, TMZ, and co-treatment, and can then be easily detected by the kit. The experiment was followed as per the manufacturer’s protocol with the LDH Cytotoxic Colorimetric Assay Kit II (BioVision, Milpitas, CA, USA). Next, we checked the apoptotic marker caspase-3. Caspase-3 is a cytosolic central activated protease found in mammalian cells which is responsible for apoptosis. It catalyzes the specific cleavage of many key cellular proteins which leads to cell death. Its presence or activation inside the cells relates to cell death via the blocking of both extrinsic and intrinsic pathways [41]. The activated caspase-3 was detected by the fluorescence plate reader as per the manufacturer’s protocol (CaspGLOW™ Fluorescein Active Caspase-3 Staining Kit, Biovision, Milpitas, CA, USA). Both the GBM cells were treated with CAP for 1 min and replaced with a recommended dose of TMZ for the specific cell. The fluorescence was recorded after 72 h of TMZ administration.

### 2.8. Definition of Control

**Figure 3:**
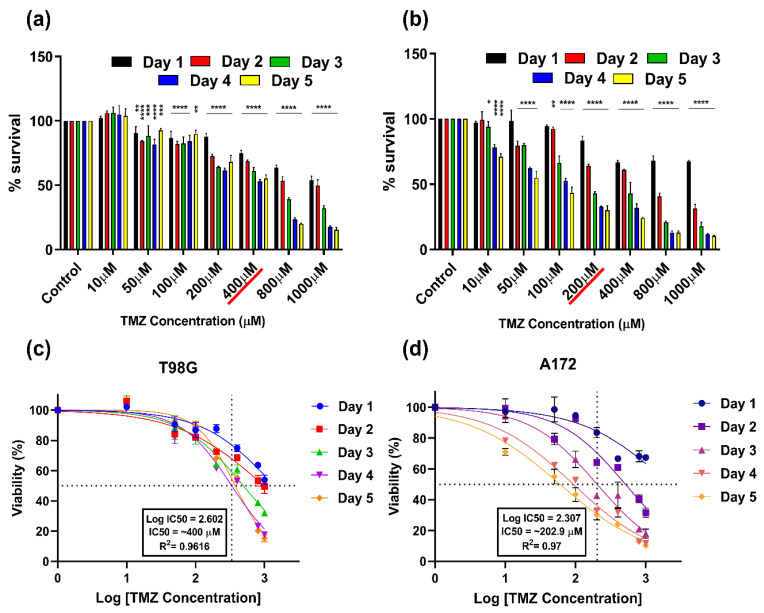
Estimation of cytotoxic dose/half-maximal inhibitory concentration (IC50) of TMZ at different doses and various time intervals for (**a**) T98G GBM cells (TMZ-resistant); (**b**) A172 GBM cells (TMZ-sensitive); (**c**) dose–response curves; the LogIC50 after 5-day TMZ treatment of T98G and A172 (**d**) cell lines. Cell viability measures were collected at 96 h (in total) after TMZ treatment. All data represent the mean ± SE, and all experiments were performed in biological triplicates. Data were analyzed using two-way ANOVA followed by Dunnett’s multiple comparisons post hoc test. All the treatment points and significance were compared to the untreated control. * *p* < 0.05; ** *p* < 0.01; *** *p* < 0.001 **** *p* < 0.0001. For each cell line n = 9. Statistical analyses are included in SI Tables S1 and S2.

**Figure 4:**
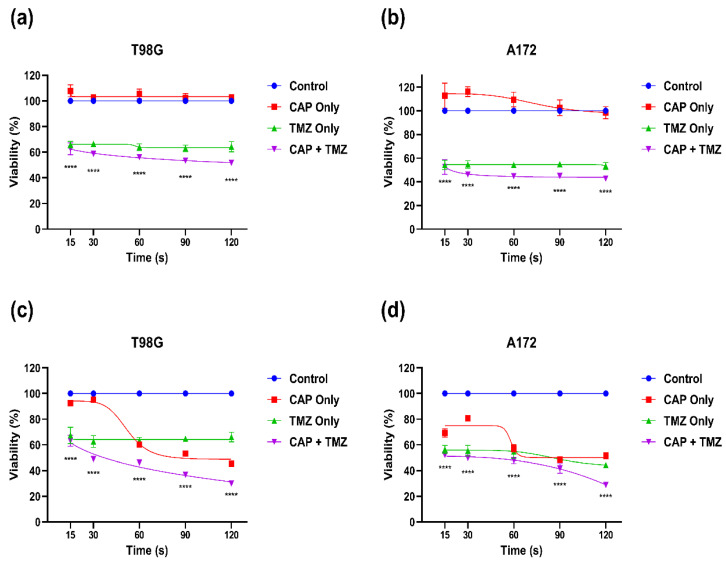
CAP sensitization phenomenon using TMZ after immediate removal of cell culture media for (**a**) T98G GBM cells and (**b**) A172 GBM cells; incubation with CAP-treated media for up to 72 h for (**c**) T98G GBM cells and (**d**) A172 GBM cells. Cell viability measures were collected at 72 h (in total) after TMZ treatment. All data represent the mean ± SE, and all experiments were performed in biological triplicates. Data were analyzed using two-way ANOVA followed by Tukey’s multiple comparisons post hoc test. All the treatment points and significance were compared to the untreated control and means of each treatment. **** *p* < 0.0001. For each cell line n = 9. Statistical analyses are included in SI Table S3.

**Figure 5:**
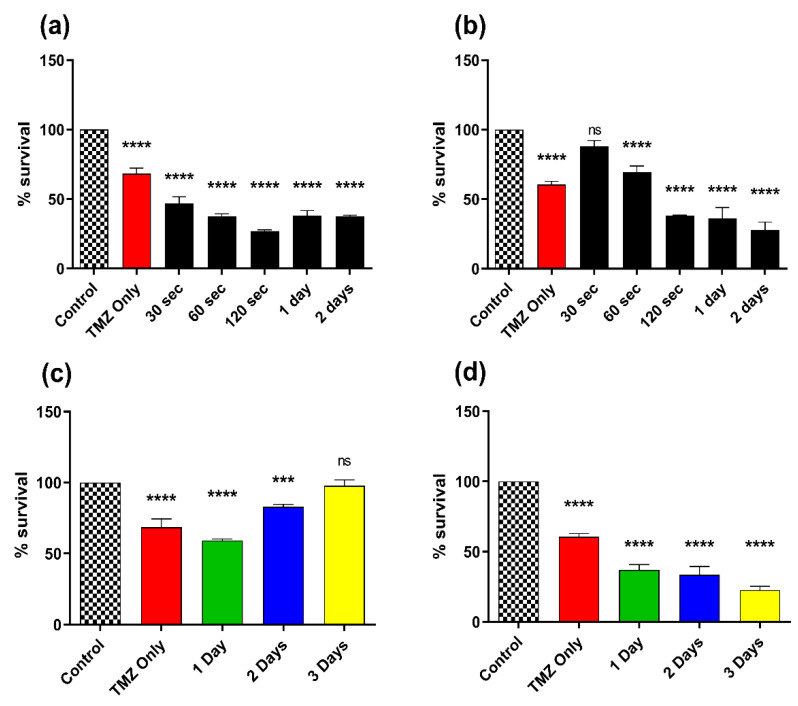
Effect of co-treatment for longer times: plasma sensitization phenomenon in the co-treatment for different incubation times for (**a**) T98G GBM cells and (**b**) A172 GBM cells; % survival after treatment with 60 s CAP treatment + TMZ on each day up to 3 days after treatment for (**c**) T98G GBM cells and (**d**) A172 GBM cells. All data represent the mean ± SE, and all experiments were performed in biological triplicates. Data were analyzed using one-way ANOVA followed by Tukey’s multiple comparisons post hoc test. All the treatment points and significance levels were compared to the untreated control and mean of each treatment. *** *p* < 0.001; **** *p* < 0.0001; ns—not significant vs. untreated control. For each cell line n = 9. Statistical analyses are included in SI Table S4.

**Figure 6:**
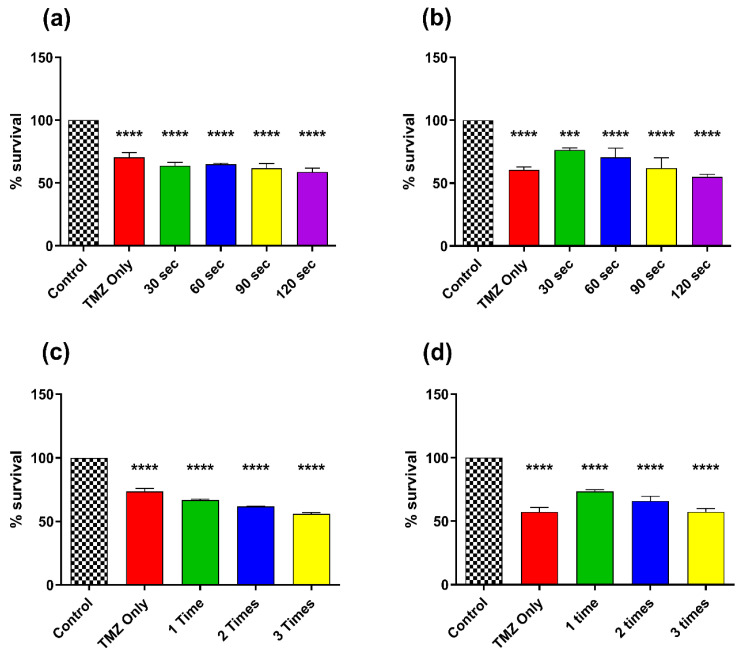
CAP sensitization phenomenon using TMZ and treatment with CAP with instant removal of cell culture media for (**a**) T98G GBM cells and (**b**) A172 GBM cells; multiple treatments with a one-time 60 s CAP jet with a 1 h gap up to 3 times for (**c**) T98G GBM cells and (**d**) A172 GBM cells. All data represent the mean ± SE, and all experiments were performed in biological triplicates. The treatment groups for each cell line were normalized to their relative untreated controls (0 s). A one-way ANOVA was performed with Tukey’s multiple comparison post hoc test. *** *p* < 0.001; **** *p* < 0.0001; ns—not significant vs. untreated control. For each cell line n = 9. Statistical analyses are included in SI Table S5.

**Figure 7:**
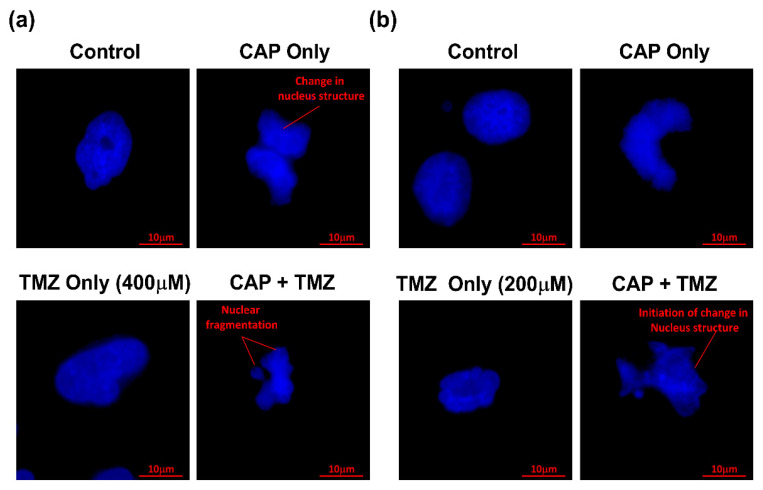
Detection of apoptosis by DAPI staining of nuclei for (**a**) T98G GBM cells and (**b**) A172 GBM cells treated by He CAP jet for 60 s in combination with TMZ. Scale bar = 10 µm. For each cell line n = 12.

**Figure 8:**
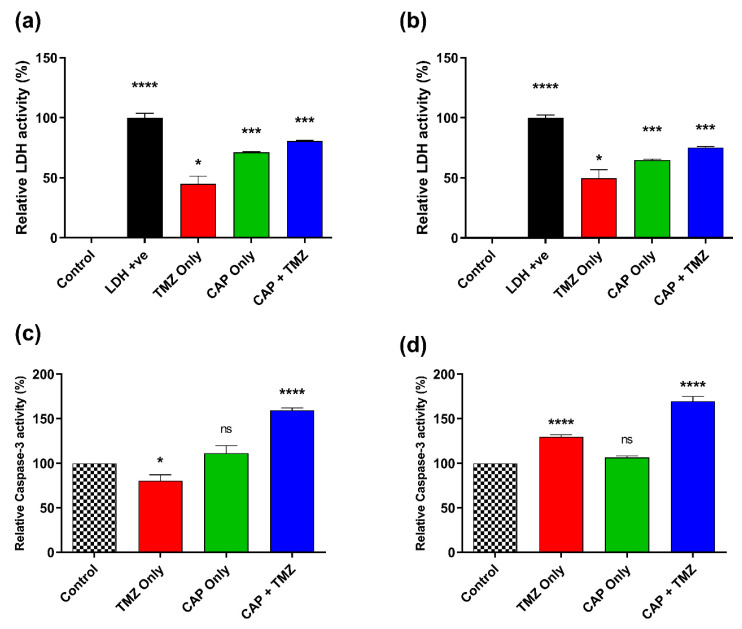
Prognostic marker and cellular damage detection by the estimation of LDH for (**a**) T98G GBM cells and (**b**) A172 GBM cells; estimation of caspase-3 levels for (**c**) T98G GBM cells and (**d**) A172 GBM cells. All data represent the mean ± SE, and experiments were performed in biological triplicates. The treatment groups for each cell line were normalized to their relative untreated controls (0 s). A one-way ANOVA was performed with Tukey’s multiple comparison post hoc test. * *p* < 0.05; *** *p* < 0.001; **** *p* < 0.0001; ns—not significant vs. untreated control. For each cell line n = 9. Statistical analyses are included in SI Table S6.

The “control” treatments in Figure 3, Figure 4, Figure 5, Figure 6, Figure 7 and Figure 8 represent that no TMZ only, CAP only and/or CAP + TMZ application were performed in these groups.

### 2.9. Statistical Analysis

For all in vitro assays, at least three experiments were performed with biological triplicates. Data were plotted using Prism 8.4.3 (GraphPad Software, San Diego, CA, USA) and are presented as the mean ± standard error. Statistical analysis was performed using one-way or two-way analysis of variance (ANOVA), as indicated. Follow-up tests were performed using Dunnett’s or Tukey’s multiple comparison post hoc test. The level of significance was denoted as follows: * *p* < 0.05; ** *p* < 0.01; *** *p* < 0.001; **** *p* < 0.0001; ns—not significant.

## 3. Results

### 3.1. CAP Jet and Characterization

As shown in Figure 2, most of the photon emissions from the plasma jet were in the UV region, as the results of N2(C3Πu) => N2(B3Πg) and N2 + (B2Σu+) => N2 + (X2Σg+) with diverse vibrational quantum numbers show, while the OH and NO peaks show the existence of RONS. Note that these UV emissions are quite important to other applications of plasma medicine, such as sterilizations. The discharge voltage represents the potential difference between the two electrodes. The streamer propagated at the peak of the voltage curve at around 18 μs, where we also found the highest discharge current peaks.

### 3.2. Cytotoxic (IC50) Dose of TMZ on T98G and A172 Cells

The cytotoxic doses (IC_50_) of TMZ-resistant (T98G) and TMZ-sensitive (A172) GBM cells were measured using an MTT assay. The survivability of both cells was estimated at different time intervals (from Day 1 to Day 5) and different concentrations of TMZ (10–1000 μM) based on previous studies [14].

In T98G cells, lower doses such as 10 μM TMZ non-significantly increased the percentage of (%) survival at all time points. However, from the 50 μM TMZ dose, the survivability started to decrease. At 50–100 μM TMZ dose, the % survival ceased ~80% on almost all days and achieved IC50 of ~400 μM on Day 4 (~49.4%). The cytotoxicity further increased in a concentration-dependent manner and reached its maximum at 1000 μM (17.62% on Day 4 and 15.2586% on Day 5) (Figure 3a). A172 GBM cells showed a different cytotoxicity pattern while receiving TMZ as compared to the T98G GBM cells. At 10 μM TMZ concentration, the cells showed little cytotoxicity on Day 2 (99.25%) and Day 3 (94.1%), while incubation up to Day 4 and Day 5 reduced survivability from 78.22% to 71.07%, respectively. The % survival decreased in further dosages of TMZ, and it reached its IC50 at ~200 μM (42.77% on Day 3; 32.81% on Day 4; 30.18% on Day 5). The cytotoxicity increased at higher TMZ concentration ranges starting from Day 2 to Day 5 (Figure 3b). The LogIC50 for T98G cells on Day 4 was found to be 2.602; (R^2^ = 0.96) = ~399.94 μM (Figure 3c). The LogIC50 of TMZ in the sensitive cell line A172 achieved 50% inhibition on Day 3 and was found to be 2.307; (R^2^ = 0.97) = ~202.9 μM (Figure 3d).

### 3.3. Combination Effect of CAP and TMZ (CAP Sensitization Phenomena)

These experiments were performed to investigate the effect of the sensitization/activation of CAP on both GBM cells. The groups were divided into four, i.e., untreated controls, CAP only, TMZ only, and CAP + TMZ. In the first experiment, GBM cells (T98G and A172) were treated at various doses of CAP, and media were removed and replaced with TMZ solution (400 μM for T98G and 200 μM for A172) immediately. In this case, there was almost no significant effect seen in T98G cells due to their resistance (Figure 4a). However, based on our initial observation, A172 cells revealed a slight increase in % cell survival after removing the CAP-treated media at 15 s of CAP treatment in some CAP-only groups, due to shorter treatment times (112.72% in CAP only; 54.24% in TMZ only, and 52.55% in CAP + TMZ group). The same pattern was observed in the 30 s CAP treatment groups as well. However, in later CAP-receiving treatment groups the % survival was comparable with the control group and caused TMZ-sensitive A172 cells to be sensitized by plasma treatment even after replacing the media and removing the possibility of RONS (Figure 4b). In both the cell lines, CAP sensitized the cells more in the co-treatment compared to either of the treatments alone.

In another set of experiments, we next investigated the combined effect of the co-treatment where CAP treatment was given and TMZ was added to it without replacing the media. This was performed to check the potential synergistic effect of the co-treatment. Interestingly, we achieved different results compared to the first set of experiments where the immediate removal of media after CAP treatment was performed. This could be a key reason for the selectivity of CAP potentially sensitizing the cells post-treatment. In this set, the TMZ dose of 400 μM did not affect the % survivability up to 72 h in T98G GBM cells. CAP treatment for 15 s and 30 s also did not show any effect on T98G GBM cells and was comparable to the control group. However, the 60 s CAP treatment significantly dropped the survivability to 60.15%, which further decreased to 53.25% (90 s) and 45.33% (120 s). The combination treatment inhibited the % survival of T98G GBM cells in a much better and more effective way compared to all other groups. The 15 s combination treatment point decreased % survival to 63.16%, which further decreased to 48.8% for 30 s, and finally to 29.90% for 120 s (Figure 4c). A172 GBM cells showed a decrease in 15 s CAP treatment in all groups, which further decreased in a CAP treatment-dependent manner and showed a maximum decrease in % survival at 120 s (28.85% in the combination group) (Figure 4d). We also compared the TMZ-only group to the CAP + TMZ groups for both cell lines to check the statistical significance between them. This also indicated that CAP can potentially sensitize GBM to TMZ. Statistical analyses are mentioned in SI Table S3.

### 3.4. Effect of Combination Therapy of CAP and TMZ in Different Parameters (for Short and Long Treatments)

To better understand the CAP sensitization phenomenon, we used different parameters of CAP treatments and their incubation with the calculated TMZ doses for both GBM cell lines. In the first set of experiments using T98G GBM cells, the incubation at short time intervals (30 s, 60 s, and 120 s) and long time intervals (1 day and 2 days) after 60 s of CAP treatment revealed interesting results. The % survival decreased when incubating the cells for up to 120 s (~27%); however, it started increasing slightly after 1-day and 2-day incubation (Figure 5a). The % survival of A172 GBM cells continued to decrease, while T98G GBM cells stabilized. In another set of experiments (Figure 5b) for A172 cells, 30 s post plasma incubation with 200 µM TMZ decreased cellular proliferation by 11.84%, while incubating for longer time intervals led to decreases in % survival of 69.58% (60 s), 38.36% (120 s), 36.05% (1 day), and 27.73% (2 day) (Figure 5b). This also indicated that longer exposure to plasma and longer incubation time with TMZ led to a decrease in % survival in sensitive cells. Interestingly, for T98G cells, TMZ alone was comparable to untreated control but did not have much inhibition effect compared to the co-treatment.

In the next set of experiments on T98G GBM cells, the incubation of TMZ with 60 s CAP treatment was performed. Here, the cells were incubated for longer time intervals and showed an initial inhibition and later slight increase in % survival at longer incubation times. At 1 day of incubation, the viability decreased as compared to the control and was noted to be 59.35%, while at 2-day and 3-day incubation intervals, it increased significantly from 83.04% to 97.83%, respectively (Figure 5c). This could be also due to the resistance of T98G cells to TMZ due to the MGMT gene. The sensitivity to A172 GBM cells appears significantly higher than compared to T98G GBM cells. Using the same parameters as mentioned above, the TMZ treatment of 200 µM led to a reduction in % survival at 1 day (37.08%) which further decreased at 2 days (33.58%) and 3 days (22.66%) (Figure 5d).

### 3.5. Effect of Combination Therapy of CAP and TMZ Using Different Parameters (for Shorter Time Intervals and Multiple Treatments)

In this set of experiments, CAP was treated for 60 s and incubated for a defined set of times (i.e., 30 s, 60 s, 90 s, and 120 s) and replaced with fresh media containing TMZ. In Figure 6a, the incubation of TMZ (400 µM) after CAP treatment incubation slightly altered the % survival of T98G GBM cells. 

The 30 s treatment point reduced % survival (63.62%). There was a slight decrease at 90 s (61.80%) with the minimum at 120 s (58.76%). However, the incubation of TMZ (200 µM) for A172 GBM cells revealed a similar but slightly higher % survival as compared to the T98G GBM cells. After 30 s of treatment the sample showed 76.31% survival, which kept on decreasing to 70.55% at 60 s, 61.96% at 90 s, and finally reached its minimum at 54.94% at 120 s (Figure 6b). This indicated that short time intervals using plasma and removing the media could possibly increase the % survival of the cells (compared to TMZ only).

In another form of experiment, the CAP dose was given one time, two times, and three times with a 1 h time gap. This result also showed very interesting outcomes as the level of % survival decreased as the number of CAP treatments increased. In T98G GBM cells, the % survival at one-time treatment was 66.79%, two times revealed 61.77%, and three times showed 55.92% (Figure 6c). However, the % survival of A172 GBM cells revealed a similar significant reduction while receiving CAP treatment one time (72.92%), which decreased for the two-time CAP treatment (65.91%) and regressed further for the three-time CAP treatment (57.54%) (Figure 6d).

### 3.6. Apoptosis Detection by DAPI Staining Using Confocal Microscopy

To confirm our results in visualized form, DAPI staining of the nucleus was performed and checked under a confocal microscope at 63X magnification. The cells were divided into four groups and were treated with CAP only, TMZ only, and the co-treatment (CAP + TMZ) vs. untreated control. T98G GBM cells in the left panel (Figure 7a) were compared with the A172 GBM cells in the right panel (Figure 7b). The induction of apoptosis in CAP-treated samples was observed and is shown by changes in the structure of the nucleus in both GBM cells. However, TMZ treatment at IC50 for both GBM cells revealed a change in nuclei structure (not round), while combination treatment clearly showed nuclear fragmentation and the initiation of the change in nucleus structure, nuclear blebbing, and chromatin condensation in both GBM cells. 

### 3.7. Effect of CAP and TMZ Combination on LDH and Caspase-3 Levels

The level of LDH within the biological system is very important in terms of the detection of tumors/cancer. LDH is released in the cell culture media when the cell membrane is damaged in necrosis or apoptosis. The results showed that TMZ-treated T98G GBM cells (400 µM) increased the LDH level up to half of the control (~44.88%) while CAP treatment further increased the level to 71.25%. The combination treatment of CAP and TMZ increased the LDH level to a maximum of up to 80.59% (Figure 8a). The LDH levels in A172 GBM cells were found to be almost identical compared to T98G GBM cells, in which TMZ treatment led to an increase in LDH concentration within the cell media of around 49.78%, which was further increased to 64.87% when receiving CAP only. The combination treatment of TMZ (200 µM) and CAP led to a higher detection of LDH levels and increased to 75.35% (Figure 8b). An increase in the levels of LDH in the combination group indicated the potential sensitization of the cells. LDH absorbance was measured at 450nm. Triplicates of each condition were used in the experiment (n = 9). Relative LDH activity (%) was noted and normalized to the controls. These results indicated that co-treatment caused more cytotoxicity to GBM cells than CAP or TMZ alone.

The level of caspase-3 in the controls was assumed to be 100%, as they were untreated samples and mean relative caspase-3 activity in the treatments was standardized and calculated based on normalized values to control. The level of caspase-3 in T98G cells in the TMZ-only treatment (400 µM) was calculated to be 80.34% due to resistance to TMZ. Its level further increased in the CAP-only treatment (111.35%). The combination of CAP + TMZ showed an increase in caspase-3 levels up to 159.41% (Figure 8c). The caspase-3 level in A172 GBM cells showed an increase in the TMZ-only group (129.65%). The CAP-only treatment showed a 106.78% increase. The combination treatment significantly increased (169.705%) caspase-3 level as compared to all other groups. This strongly suggests the activation of apoptosis in the combination group of A172 GBM cells (Figure 8d). Both the cell types attained higher caspase-3 levels, indicating plasma- and TMZ-induced apoptosis.

## 4. Discussion

Chemotherapy is one of the popular therapies used to treat cancer. Various chemotherapeutic drugs are discovered and used to date that specifically act on cancer cells [5]. Temozolomide is a known standard chemotherapeutic drug used for the treatment of glioblastoma [42]. However, the long-term survival rate of glioblastoma patients is very low after receiving TMZ treatment because of drug resistance [43], and hence, there is an urgent need for a strategy to increase the efficacy of TMZ. On the other hand, enhancing efficacy can lead to reduced toxicity by reducing the TMZ dose. In the past few years, many studies have focused on the use of CAP to treat various kinds of cancers, including glioblastoma. These studies have indicated that CAP is a promising approach to inhibit and kill cancerous cells selectively [14,36]. CAP treatment induces apoptosis and inhibits the cell cycle, alternate cell survival pathway, and autophagy [44,45]. Recent studies have reported that CAP can kill cancer cells by initiating apoptosis, necrosis, and autophagy, which eventually leads to cell death [46]. There are different gases that can be used to generate CAP, such as helium, argon, nitrogen, and air. Indeed, multiple ways to generate plasmas involve techniques such as corona discharge, dielectric barrier discharge, and plasma jets at atmospheric pressure and low temperature. These devices may have different applications in agriculture, disinfection/sterilization, wound healing, dentistry, and in medicine for treatment of bacteria, viruses, and cancer [47]. Combination therapy is a new modality of treatment used to enhance the primary effect of the drug and potentially sensitize the cell’s response to the treatment. We investigated the role of CAP and TMZ as a co-treatment. The co-therapy of CAP with many chemical moieties and nanoparticles has been successfully used in the treatment of various cancers in in vitro and in vivo models [48]. Here, we used artificially produced CAP by using He gas which generates ionization and the production of electrons, ions, neutrals, UV light, and reactive oxygen and nitrogen species (RONS).

In the past decade, there have been multiple combination treatments used with TMZ in several clinical trials for high-grade GBM patients. These therapies include chemotherapy, radiotherapy, gene therapy, microRNA, and immunotherapy. However, due to various resistance factors, these co-treatments are limited. One such resistance is due to the MGMT gene promoter, as discussed earlier. TMZ alone is not effective in treating GBM in MGMT-positive patients due to repairs [49]. Hence, combinations of various adjuvant therapies have been used to treat GBM. Another important aspect in treating GBM is the ability of a drug to cross the blood–brain barrier. These limitations make the treatment of GBM difficult. Conventional GBM treatment includes surgical resection followed by adjuvant radiotherapy. In a classic study, Stupp et al. compared the efficacy of radiotherapy alone with the combination of radiotherapy plus TMZ [50,51,52]. Both of which have their drawbacks and have significant side effects. In our previous investigation, we showed that a single non-invasive application of a CAP jet for 1 min followed by TMZ administration caused inhibition in brain tumor growth in the U-87MG xenograft intracranial murine model [14]. In the last decade, in vivo models have been developed to understand the biology of stem cells and how they have a critical role in the development, metastasis, and recurrence of tumors. The recurrence of GBM in clinical models is largely associated with the relapse of tumors from stem cells after initial treatment. Thus, targeting stem cells in GBM is an extremely important aspect of the clinical treatment of GBM. The use of CAP in the treatment of GBM is one proposed mechanism that may increase ROS concentration in the tumor and inhibit it [22]. Indeed, we and others have shown that tumor-treating fields (TTFs) have been shown to significantly inhibit the tumor in vitro and in vivo [53,54,55]. Multiple clinical trials investigating the use of CAP and adjuvant therapy for patients with GBM are currently ongoing. However, the current data demonstrate a potential sensitization effect and the preclinical effectiveness of the CAP + TMZ combination in vitro. In line with our findings using CAP application in vitro, combination treatment was effective in sensitizing GBM to TMZ.

Earlier studies have revealed a plausible CAP effect in cell viability inhibition. The latest study also states that CAP treatment with TMZ at a certain dose can inhibit U-87MG brain cancer cells [14,40]. In the present study, the cytotoxicity of the TMZ dose was calculated revealing the IC50 of TMZ-resistant cell line (T98G) to be ~ 400 µM and the TMZ-sensitive (A172) GBM cells to be ~200 µM. The response of various TMZ-resistant brain cancer cells could be attributed to the overexpression of a gene named O6-methylguanine methyltransferase (MGMT). T98G cells are more TMZ-resistant compared to A172 cells due to the over-expression of MGMT and an increase in the base excision repair system [49]. However, in most cases, the MGMT gene remains silent, and GBM remains incurable. As stated in previous papers, CAP induces many short-lived reactive species which may take part in cell death if these species persist in the media/system for a longer time [56]. In Figure 4a, the immediate removal of cell media after CAP treatment did not alter GBM’s survivability, as T98G cells are more resistant. Although, in TMZ-sensitive A172 GBM cells (Figure 4b) at a lower dose, the cancer cells started proliferating slightly; this ceased after longer treatments and was comparable to the control. This could have been due to the presence of CAP-generated short-lived reactive species OH·- and O_2_-, which react with each other to form stable reactive species [57], but as the media was taken out immediately, the effect was not significantly visible in either GBM type. However, the effect of long-lived reactive species (i.e., H_2_O_2_) could be clearly seen in both GBMs when the media were not removed (Figure 4c,d). 

The cell sensitization/activation phenomena of CAP-induced RONS and electromagnetic radiation were also revealed in further experiments where different conditions were used to treat GBM cells. TMZ-resistant T98G GBM cell viability was decreased when the cells were treated with CAP plus TMZ at lower time intervals up to 120 s, while TMZ-sensitive A172 GBM cells showed sensitization and started regression at later time intervals. Untreated controls were not affected as they did not receive any treatments. CAP is very well-known to induce many short- and long-lived reactive species. The presence of short-lived species in CAP may help TMZ sensitive cells to grow more quickly as compared to TMZ-resistant cells. We define this as the potential sensitization phenomenon of the cells with CAP treatment, in which, at lower concentrations, cancer cells are sensitized and begin to proliferate or die (without TMZ), but cell death increases at later and longer time intervals due to the accumulation of reactive species [58]. 

A noticeable trend of increase in cell survival (compared to the TMZ group) was observed in A172 GBM cells at lower CAP doses in all experimental conditions, which regressed later in the CAP treatment groups with longer times. This may be due to the activation of the MGMT gene in A172 GBM cells which promotes cellular proliferation when combined with TMZ at a lower dose of CAP [49]. CAP mainly kills cancer cells by apoptosis, and here it was visualized by DAPI staining using confocal microscopy [59]. The effect of CAP-induced RONS leads to DNA damage and activates various cell death pathways, i.e., HEF-cMET, PI3K-AKT, etc., and results in apoptosis [14,23,24]. Here, Figure 7a,b revealed a change in the structure of the nucleus in CAP-treated cells. The combination group showed intense damage and exhibited nuclear fragmentation and chromatin condensation, a sign of apoptosis which could be due to the plausible reasons discussed above.

Lactate dehydrogenase (LDH) enzyme is secreted when cells are under great stress, and its presence directly correlates with the presence of cancer cells [60]. The LDH enzyme is responsible for the conversion of pyruvate to lactate in cellular processes and gives energy to cancer cells [61]. The combined effect of CAP and TMZ leads to the generation of OH· radical, which is the main reactive species responsible for the inactivation of LDH activity [62]. Irrespective of this, co-treatment application to the cells is sufficient to increase LDH levels in the media to relate to a decrease in GBM cells as shown in Figure 8a,b. Caspase-3 is a gene responsible for apoptosis and is activated when DNA damage occurs due to the activation of PARP and other related genes [26]. Figure 8c,d reveals that CAP alone increased the caspase-3 activity at a very low level, while the combination treatment with TMZ increased the caspase-3 activity significantly compared with all related groups. This clearly suggests that combination treatment works much better and can induce DNA damage in both types of GBM cells, which is consistent with our previous results. It is also indicative of the potential sensitization of the cells caused by CAP treatment, especially in the TMZ-resistant T98G cells that did not respond well to TMZ before CAP treatment. 

## 5. Conclusions

Collectively, our study has demonstrated potential sensitization effects using short and long doses of plasma, as well as the efficacy of TMZ alone and in combination with CAP on two different GBM cell lines. Various strategies were implemented to confirm the basic questions about overcoming resistance to chemotherapeutic drugs (TMZ) by CAP treatment in GBM cells in a better way. The TMZ-resistant T98G GBM cells worked well in lower CAP doses as compared to TMZ-sensitive A172 GBM cells, which could be attributed to the MGMT gene responsible for the resistance of these cells to TMZ. The short-lived reactive species at lower CAP treatment times induced better inhibition in T98G GBM cells, while the opposite was found in A172 GBM cells where the cells were sensitized and tended to slightly proliferate. The activation of apoptosis and caspase-3 clearly showed that combination therapy worked better to treat both GBM cell types. Hence, these results indicate that pre-treatment with CAP could possibly sensitize GBM to chemotherapy and give a better response. Furthermore, the CAP + TMZ combination leads to a new paradigm in cancer therapy in vitro, which can also be applied in vivo by offering a non-invasive surgical modality without affecting the surrounding tissue to enhance the effect of TMZ in animal models.

## Data Availability

The data presented in this study are available on request from the corresponding author.

## Supplementary Materials

The following supporting information can be downloaded at: https://www.mdpi.com/article/10.3390/cancers14133116/s1, Table S1–S6: Statistical analyses for Figure 3, Figure 4, Figure 5, Figure 6 and Figure 8.

## References

[1] Persaud-Sharma D., Burns J., Trangle J., Moulik S. (2017). Disparities in Brain Cancer in the United States: A Literature Review of Gliomas. Med. Sci..

[2] Hayat M.A. (2012). Tumors of the Central Nervous System.

[3] Shah V., Kochar P. (2018). Brain Cancer: Implication to Disease, Therapeutic Strategies and Tumor Targeted Drug Delivery Approaches. Recent Pat. Anti-Cancer Drug Discov..

[4] Siegel R.L., Miller K.D., Jemal A. (2020). Cancer statistics, 2020. CA Cancer J. Clin..

[5] Laquintana V., Trapani A., Denora N., Wang F., Gallo J.M., Trapani G. (2009). New strategies to deliver anticancer drugs to brain tumors. Expert Opin. Drug Deliv..

[6] Harder B.G., Blomquist M.R., Wang J., Kim A.J., Woodworth G.F., Winkles J.A., Loftus J.C., Tran N.L. (2018). Developments in Blood-Brain Barrier Penetrance and Drug Repurposing for Improved Treatment of Glioblastoma. Front. Oncol..

[7] Shergalis A., Bankhead A., Luesakul U., Muangsin N., Neamati N. (2018). Current Challenges and Opportunities in Treating Glioblastoma. Pharmacol. Rev..

[8] Carter T.C., Medina-Flores R., Lawler B.E. (2018). Glioblastoma Treatment with Temozolomide and Bevacizumab and Overall Survival in a Rural Tertiary Healthcare Practice. BioMed Res. Int..

[9] Strobel H., Baisch T., Fitzel R., Schilberg K., Siegelin M.D., Karpel-Massler G., Debatin K.-M., Westhoff M.-A. (2019). Temozolomide and Other Alkylating Agents in Glioblastoma Therapy. Biomedicines.

[10] Zhang J., Stevens M.F., Bradshaw T.D. (2012). Temozolomide: Mechanisms of Action, Repair and Resistance. Curr. Mol. Pharmacol..

[11] Hegi M.E., Diserens A.-C., Gorlia T., Hamou M.-F., De Tribolet N., Weller M., Kros J.M., Hainfellner J.A., Mason W., Mariani L. (2005). MGMT Gene Silencing and Benefit from Temozolomide in Glioblastoma. N. Engl. J. Med..

[12] Woo P.Y., Li Y., Chan A.H., Ng S.C., Loong H.H., Chan D.T., Wong G.K., Poon W.S. (2019). A multifaceted review of temozolomide resistance mechanisms in glio-blastoma beyond O-6-methylguanine-DNA methyltransferase. Glioma.

[13] Yi G.-Z., Huang G., Guo M., Zhang X., Wang H., Deng S., Li Y., Xiang W., Chen Z., Pan J. (2019). Acquired temozolomide resistance in MGMT-deficient glioblastoma cells is associated with regulation of DNA repair by DHC2. Brain.

[14] Soni V., Adhikari M., Simonyan H., Lin L., Sherman J.H., Young C.N., Keidar M. (2021). In Vitro and In Vivo Enhancement of Temozolomide Effect in Human Glioblastoma by Non-Invasive Application of Cold Atmospheric Plasma. Cancers.

[15] Keidar M., Shashurin A., Volotskova O., Stepp M.A., Srinivasan P., Sandler A., Trink B. (2013). Cold atmospheric plasma in cancer therapy. Phys. Plasmas.

[16] Keidar M., Yan D., Beilis I.I., Trink B., Sherman J.H. (2018). Plasmas for Treating Cancer: Opportunities for Adaptive and Self-Adaptive Approaches. Trends Biotechnol..

[17] Bernhardt T., Semmler M.L., Schäfer M., Bekeschus S., Emmert S., Boeckmann L. (2019). Plasma Medicine: Applications of Cold Atmospheric Pressure Plasma in Dermatology. Oxidative Med. Cell. Longev..

[18] Xiong Z. (2018). Cold Atmospheric Pressure Plasmas (COLD PLASMAs) for Skin Wound Healing.

[19] Sakudo A., Yagyu Y., Onodera T. (2019). Disinfection and Sterilization Using Plasma Technology: Fundamentals and Future Perspectives for Biological Applications. Int. J. Mol. Sci..

[20] Adhikari B., Adhikari M., Ghimire B., Park G., Choi E.H. (2019). Cold Atmospheric Plasma-Activated Water Irrigation Induces Defense Hormone and Gene expression in Tomato seedlings. Sci. Rep..

[21] Adhikari B., Adhikari M., Ghimire B., Adhikari B.C., Park G., Choi E.H. (2020). Cold plasma seed priming modulates growth, redox homeostasis and stress response by inducing reactive species in tomato (*Solanum lycopersicum*). Free Radic. Biol. Med..

[22] Keidar M. (2018). A prospectus on innovations in the plasma treatment of cancer. Phys. Plasmas.

[23] Adhikari M., Kaushik N., Ghimire B., Adhikari B., Baboota S., Al-Khedhairy A.A., Wahab R., Lee S.-J., Kaushik N.K., Choi E.H. (2019). Cold atmospheric plasma and silymarin nanoemulsion synergistically inhibits human melanoma tumorigenesis via targeting HGF/c-MET downstream pathway. Cell Commun. Signal..

[24] Adhikari M., Adhikari B., Adhikari A., Yan D., Soni V., Sherman J., Keidar M. (2020). Cold Atmospheric Plasma as a Novel Therapeutic Tool for the Treatment of Brain Cancer. Curr. Pharm. Des..

[25] Akter M., Jangra A., Choi S.A., Choi E.H., Han I. (2020). Non-Thermal Atmospheric Pressure Bio-Compatible Plasma Stimulates Apoptosis via p38/MAPK Mechanism in U87 Malignant Glioblastoma. Cancers.

[26] Adhikari M., Adhikari B., Kaushik N., Lee S.-J., Kaushik N.K., Choi E.H. (2019). Melanoma Growth Analysis in Blood Serum and Tissue Using Xenograft Model with Response to Cold Atmospheric Plasma Activated Medium. Appl. Sci..

[27] Gjika E., Pal-Ghosh S., Tang A., Kirschner M., Tadvalkar G., Canady J., Stepp M.A., Keidar M. (2018). Adaptation of Operational Parameters of Cold Atmospheric Plasma for in Vitro Treatment of Cancer Cells. ACS Appl. Mater. Interfaces.

[28] Adhikari M., Adhikari B., Ghimire B., Baboota S., Choi E.H. (2020). Cold Atmospheric Plasma and Silymarin Nanoemulsion Activate Autophagy in Human Melanoma Cells. Int. J. Mol. Sci..

[29] Fridman G., Friedman G., Gutsol A., Shekhter A.B., Vasilets V.N., Fridman A. (2008). Applied Plasma Medicine. Plasma Process. Polym..

[30] Dikalov S.I., Harrison D.G. (2014). Methods for Detection of Mitochondrial and Cellular Reactive Oxygen Species. Antioxid. Redox Signal..

[31] Leduc M., Guay D., Coulombe S., Leask R.L. (2010). Effects of Non-thermal Plasmas on DNA and Mammalian Cells. Plasma Process. Polym..

[32] Yao X., Lin L., Soni V., Gjika E., Sherman J.H., Yan D., Keidar M. (2020). Sensitization of glioblastoma cells to temozolomide by a helium gas discharge tube. Phys. Plasmas.

[33] Shashurin A., Keidar M., Bronnikov S., Jurjus R.A., Stepp M.A. (2008). Living tissue under treatment of cold plasma atmospheric jet. Appl. Phys. Lett..

[34] Zucker S.N., Zirnheld J., Bagati A., Disanto T.M., Soye B.D., Wawrzyniak J.A., Etemadi K., Nikiforov M., Berezney R. (2012). Preferential induction of apoptotic cell death in melanoma cells as compared with normal keratinocytes using a non-thermal plasma torch. Cancer Biol. Ther..

[35] Iseki S., Nakamura K., Hayashi M., Tanaka H., Kondo H., Kajiyama H., Kano H., Kikkawa F., Hori M. (2012). Selective killing of ovarian cancer cells through induction of apoptosis by nonequilibrium atmospheric pressure plasma. Appl. Phys. Lett..

[36] Kaushik N.K., Kaushik N., Yoo K.C., Uddin N., Kim J.S., Lee S.J., Choi E.H. (2016). Low doses of PEG-coated gold nanoparticles sensitize solid tumors to cold plasma by blocking the PI3K/AKT-driven signaling axis to suppress cellular transformation by inhibiting growth and EMT. Biomaterials.

[37] Li W., Yu H., Ding D., Chen Z., Wang Y., Wang S., Li X., Keidar M., Zhang W. (2018). Cold atmospheric plasma and iron oxide-based magnetic nanoparticles for synergetic lung cancer therapy. Free Radic. Biol. Med..

[38] Zhu W., Lee S.-J., Castro N.J., Yan D., Keidar M., Zhang L.G. (2016). Synergistic Effect of Cold Atmospheric Plasma and Drug Loaded Core-shell Nanoparticles on Inhibiting Breast Cancer Cell Growth. Sci. Rep..

[39] Young R.M., Jamshidi A., Davis G., Sherman J.H. (2015). Current trends in the surgical management and treatment of adult glioblastoma. Ann. Transl. Med..

[40] Gjika E., Pal-Ghosh S., Kirschner M.E., Lin L., Sherman J.H., Stepp M.A., Keidar M. (2020). Combination therapy of cold atmospheric plasma (CAP) with temozolomide in the treatment of U87MG glioblastoma cells. Sci. Rep..

[41] Krautwald S., Ziegler E., Rölver L., Linkermann A., Keyser K.A., Steen P., Wollert K.C., Klingebiel M.K., Kunzendorf U. (2010). Effective Blockage of Both the Extrinsic and Intrinsic Pathways of Apoptosis in Mice by TAT-crmA. J. Biol. Chem..

[42] Bahadur S., Sahu A.K., Baghel P., Saha S. (2019). Current promising treatment strategy for glioblastoma multiform: A review. Oncol. Rev..

[43] Zhu P., Du X.L., Lu G., Zhu J.-J. (2017). Survival benefit of glioblastoma patients after FDA approval of temozolomide concomitant with radiation and bevacizumab: A population-based study. Oncotarget.

[44] Fridman G., Shereshevsky A., Jost M.M., Brooks A.D., Fridman A., Gutsol A., Vasilets V., Friedman G. (2007). Floating Electrode Dielectric Barrier Discharge Plasma in Air Promoting Apoptotic Behavior in Melanoma Skin Cancer Cell Lines. Plasma Chem. Plasma Process..

[45] Shi L., Ito F., Wang Y., Okazaki Y., Tanaka H., Mizuno M., Hori M., Hirayama T., Nagasawa H., Richardson D.R. (2017). Non-thermal plasma induces a stress response in mesothelioma cells resulting in increased endocytosis, lysosome biogenesis and autophagy. Free Radic. Biol. Med..

[46] Semmler M.L., Bekeschus S., Schäfer M., Bernhardt T., Fischer T., Witzke K., Seebauer C., Rebl H., Grambow E., Vollmar B. (2020). Molecular Mechanisms of the Efficacy of Cold Atmospheric Pressure Plasma (CAP) in Cancer Treatment. Cancers.

[47] Domonkos M., Tichá P., Trejbal J., Demo P. (2021). Applications of Cold Atmospheric Pressure Plasma Technology in Medicine, Agriculture and Food Industry. Appl. Sci..

[48] Gao L., Shi X., Wu X. (2020). Applications and challenges of low temperature plasma in pharmaceutical field. J. Pharm. Anal..

[49] Lee S.Y. (2016). Temozolomide resistance in glioblastoma multiforme. Genes Dis..

[50] Stupp R., Mason W.P., van den Bent M.J., Weller M., Fisher B., Taphoorn M.J.B., Belanger K., Brandes A.A., Marosi C., Bogdahn U. (2005). Radiotherapy plus Concomitant and Adjuvant Temozolomide for Glioblastoma. N. Engl. J. Med..

[51] Parisi S., Corsa P., Raguso A., Perrone A., Cossa S., Munafò T., Sanpaolo G., Donno E., Clemente M.A., Piombino M. (2015). Temozolomide and Radiotherapy versus Radiotherapy Alone in High Grade Gliomas: A Very Long Term Comparative Study and Literature Review. BioMed Res. Int..

[52] Combs S.E., Gutwein S., Schulz-Ertner D., van Kampen M., Thilmann C., Edler L., Wannenmacher M.M., Debus J. (2005). Temozolomide Combined with Irradiation as Postoperative Treatment of Primary Glioblastoma Multiforme. Strahlenther. und Onkol..

[53] Yao X., Goldstein I., Lin L., Sherman J.H., Keidar M. (2020). Comparative Study of Cancer Treatment Potential Effects of Tumor-Treating Fields and Cold Atmospheric Plasma. Plasma Med..

[54] Rominiyi O., Vanderlinden A., Clenton S.J., Bridgewater C., Al-Tamimi Y., Collis S.J. (2020). Tumour treating fields therapy for glioblastoma: Current advances and future directions. Br. J. Cancer.

[55] Carrieri F.A., Smack C., Siddiqui I., Kleinberg L.R., Tran P.T. (2020). Tumor Treating Fields: At the Crossroads Between Physics and Biology for Cancer Treatment. Front. Oncol..

[56] Kim S.J., Chung T.H. (2016). Cold atmospheric plasma jet-generated RONS and their selective effects on normal and carcinoma cells. Sci, Rep..

[57] Bauer G. (2019). The synergistic effect between hydrogen peroxide and nitrite, two long-lived molecular species from cold atmospheric plasma, triggers tumor cells to induce their own cell death. Redox Biol..

[58] Köritzer J., Boxhammer V., Schäfer A., Shimizu T., Klämpfl T.G., Li Y.-F., Welz C., Schwenk-Zieger S., Morfill G.E., Zimmermann J.L. (2013). Restoration of Sensitivity in Chemo—Resistant Glioma Cells by Cold Atmospheric Plasma. PLoS ONE.

[59] Yadav D.K., Adhikari M., Kumar S., Ghimire B., Han I., Kim M.-H., Choi E.-H. (2020). Cold atmospheric plasma generated reactive species aided inhibitory effects on human melanoma cells: An in vitro and in silico study. Sci. Rep..

[60] Palmer S.R., Erickson L.A., Ichetovkin I., Knauer D.J., Markovic S.N. (2011). Circulating Serologic and Molecular Biomarkers in Malignant Melanoma. Mayo Clin. Proc..

[61] Goldman R.D., O’Kaplan N., Hall T.C. (1964). Lactic Dehydrogenase in Human Neoplastic Tissues. Cancer Res..

[62] Buchanan J.D., Armstrong D. (1976). Free radical inactivation of lactate dehydrogenase. Int. J. Radiat. Biol. Relat. Stud. Phys. Chem. Med..

