# Supplementary material for: Theranostic Potential of Adaptive Cold Atmospheric Plasma with Temozolomide to Checkmate Glioblastoma: An In Vitro Study
Source: Cancers (Basel). 2022 Jun 25;14(13):3116. doi: 10.3390/cancers14133116 (PMC9264842; doi:10.3390/cancers14133116)
Supplement: Supplementary file 1 [file cancers-14-03116-s001.zip › cancers-1759453-supplementary.pdf]

Theranostic Potential of Adaptive Cold Atmospheric Plasma with Temozolomide to Checkmate Glioblastoma: An In Vitro Study

Vikas Soni, Manish Adhikari, Li Lin, Jonathan H. Sherman and Michael Keidar

Table S1. Statistical analyses for Figures 3(a,c) IC50 of TMZ for T98G cells; A two-way ANOVA followed by Dunnett's multiple comparisons post hoc tests were performed. All the treatment points and significance were compared to untreated control. * p < 0.05; ** p < 0.01; and **** p < 0.0001, ns—not significant vs. untreated control. n = 9.

Dunnett's multiple comparisons test	Mean Diff.	95.00% CI of diff.	Significant?	Summary
T98G Cells: IC50 of TMZ				
Day 1				
Control vs. 10µM	-2.092	-9.086 to 4.902	No	ns
Control vs. 50µM	9.58	2.586 to 16.57	Yes	**
Control vs. 100µM	13.27	6.278 to 20.27	Yes	****
Control vs. 200µM	12.36	5.366 to 19.35	Yes	****
Control vs. 400µM	25.09	18.10 to 32.09	Yes	****
Control vs. 800µM	36.38	29.39 to 43.37	Yes	****
Control vs. 1000µM	46.1	39.10 to 53.09	Yes	****
Day 2				
Control vs. 10µM	-5.933	-12.93 to 1.061	No	ns
Control vs. 50µM	15.97	8.979 to 22.97	Yes	****
Control vs. 100µM	17.91	10.91 to 24.90	Yes	****
Control vs. 200µM	27.48	20.49 to 34.48	Yes	****
Control vs. 400µM	31.49	24.49 to 38.48	Yes	****
Control vs. 800µM	46.7	39.71 to 53.70	Yes	****
Control vs. 1000µM	50.33	43.34 to 57.33	Yes	****
Day 3				
Control vs. 10µM	-6.026	-13.02 to 0.9684	No	ns
Control vs. 50µM	11.55	4.556 to 18.54	Yes	***
Control vs. 100µM	17.5	10.51 to 24.49	Yes	****
Control vs. 200µM	35.86	28.86 to 42.85	Yes	****
Control vs. 400µM	39.11	32.12 to 46.11	Yes	****
Control vs. 800µM	61.07	54.07 to 68.06	Yes	****
Control vs. 1000µM	67.87	60.88 to 74.87	Yes	****
Day 4				
Control vs. 10µM	-5.121	-12.11 to 1.873	No	ns
Control vs. 50µM	18.3	11.31 to 25.30	Yes	****
Control vs. 100µM	15.64	8.648 to 22.64	Yes	****
Control vs. 200µM	38.44	31.44 to 45.43	Yes	****
Control vs. 400µM	46.99	40.00 to 53.98	Yes	****

Control vs. 800μM	76.75	69.75 to 83.74	Yes	****
Control vs. 1000μM	82.37	75.38 to 89.37	Yes	****
Day 5				
Control vs. 10μM	-3.836	-10.83 to 3.158	No	ns
Control vs. 50μM	7.144	0.1495 to 14.14	Yes	*
Control vs. 100μM	10.24	3.247 to 17.24	Yes	**
Control vs. 200μM	31.8	24.81 to 38.79	Yes	****
Control vs. 400μM	44.66	37.67 to 51.66	Yes	****
Control vs. 800μM	79.92	72.93 to 86.92	Yes	****
Control vs. 1000μM	84.74	77.75 to 91.74	Yes	****

Table S2. Statistical analyses for Figures 3(b,d) IC50 of TMZ for A172 cells; A two-way ANOVA followed by Dunnett's multiple comparisons post hoc tests were performed. All the treatment points and significance were compared to untreated control. * $p < 0.05$; ** $p < 0.01$; and **** $p < 0.0001$, ns—not significant vs. untreated control. n = 9.

Dunnett's multiple comparisons test	Mean Diff.	95.00% CI of diff.	Significant?	Summary
A172 Cells: IC50 of TMZ				
Day 1				
Control vs. 10μM	2.983	-2.894 to 8.860	No	ns
Control vs. 50μM	1.484	-4.393 to 7.361	No	ns
Control vs. 100μM	5.45	-0.4270 to 11.33	No	ns
Control vs. 200μM	16.49	10.62 to 22.37	Yes	****
Control vs. 400μM	33.35	27.48 to 39.23	Yes	****
Control vs. 800μM	31.76	25.89 to 37.64	Yes	****
Control vs. 1000μM	32.56	26.68 to 38.44	Yes	****
Day 2				
Control vs. 10μM	0.7439	-5.133 to 6.621	No	ns
Control vs. 50μM	20.65	14.77 to 26.53	Yes	****
Control vs. 100μM	7.546	1.669 to 13.42	Yes	**
Control vs. 200μM	35.89	30.01 to 41.77	Yes	****
Control vs. 400μM	39.08	33.21 to 44.96	Yes	****
Control vs. 800μM	59.41	53.53 to 65.29	Yes	****
Control vs. 1000μM	68.42	62.55 to 74.30	Yes	****
Day 3				
Control vs. 10μM	5.899	0.02210 to 11.78	Yes	*
Control vs. 50μM	20.05	14.17 to 25.93	Yes	****
Control vs. 100μM	33.73	27.85 to 39.60	Yes	****
Control vs. 200μM	57.22	51.34 to 63.10	Yes	****
Control vs. 400μM	57.13	51.25 to 63.00	Yes	****
Control vs. 800μM	78.96	73.09 to 84.84	Yes	****
Control vs. 1000μM	82.03	76.15 to 87.91	Yes	****
Day 4				
Control vs. 10μM	21.78	15.90 to 27.65	Yes	****

Control vs. 50μM	37.71	31.83 to 43.59	Yes	****
Control vs. 100μM	47.3	41.43 to 53.18	Yes	****
Control vs. 200μM	67.19	61.31 to 73.07	Yes	****
Control vs. 400μM	68.21	62.33 to 74.08	Yes	****
Control vs. 800μM	87.2	81.32 to 93.08	Yes	****
Control vs. 1000μM	88.49	82.62 to 94.37	Yes	****
Day 5				
Control vs. 10μM	28.93	23.05 to 34.81	Yes	****
Control vs. 50μM	44.91	39.04 to 50.79	Yes	****
Control vs. 100μM	56.65	50.78 to 62.53	Yes	****
Control vs. 200μM	69.81	63.93 to 75.69	Yes	****
Control vs. 400μM	75.82	69.95 to 81.70	Yes	****
Control vs. 800μM	87.1	81.23 to 92.98	Yes	****
Control vs. 1000μM	89.76	83.88 to 95.64	Yes	****

Table S3. Statistical analyses for Figures 4(a-d) for both the cell lines; Comparison of treatments between TMZ only and CAP + TMZ groups. A two-way ANOVA followed by Tukey's multiple comparisons post hoc tests were performed. All the treatment points and significance were compared to untreated control. * $p < 0.05$; ** $p < 0.01$; and **** $p < 0.0001$, ns—not significant vs. untreated control. n = 9.

Figure 4(a): T98G cells

Treatment Time (s)	Tukey's multiple comparisons test	Summary	Adjusted P Value	Mean Diff.
15 Sec	TMZ Only vs. CAP + TMZ	ns	0.3911	3.212
30 Sec	TMZ Only vs. CAP + TMZ	**	0.002	7.814
60 Sec	TMZ Only vs. CAP + TMZ	**	0.0021	7.789
90 Sec	TMZ Only vs. CAP + TMZ	***	0.0001	9.621
120 Sec	TMZ Only vs. CAP + TMZ	****	<0.0001	12.61

Figure 4(b): A172 cells

Treatment Time (s)	Tukey's multiple comparisons test	Summary	Adjusted P Value	Mean Diff.
15 Sec	TMZ Only vs. CAP + TMZ	ns	0.9567	1.686
30 Sec	TMZ Only vs. CAP + TMZ	ns	0.0689	8.428
60 Sec	TMZ Only vs. CAP + TMZ	*	0.0319	9.519
90 Sec	TMZ Only vs. CAP + TMZ	*	0.0212	10.07
120 Sec	TMZ Only vs. CAP + TMZ	*	0.0145	10.55

Figure 4(c): T98G cells

Treatment Time (s)	Tukey's multiple comparisons test	Summary	Adjusted P Value	Mean Diff.
15 Sec	TMZ Only vs. CAP + TMZ	ns	0.1547	4.193
30 Sec	TMZ Only vs. CAP + TMZ	****	<0.0001	13.82
60 Sec	TMZ Only vs. CAP + TMZ	****	<0.0001	17.02
90 Sec	TMZ Only vs. CAP + TMZ	****	<0.0001	28.01
120 Sec	TMZ Only vs. CAP + TMZ	****	<0.0001	36.19

Figure 4(d): A172 cells

Treatment Time (s)	Tukey's multiple comparisons test	Summary	Adjusted P Value	Mean Diff.
15 Sec	TMZ Only vs. CAP + TMZ	ns	0.1391	4.244
30 Sec	TMZ Only vs. CAP + TMZ	*	0.0242	5.733
60 Sec	TMZ Only vs. CAP + TMZ	**	0.0042	6.99
90 Sec	TMZ Only vs. CAP + TMZ	**	0.0031	7.189
120 Sec	TMZ Only vs. CAP + TMZ	****	<0.0001	15.49

Table S4. Statistical analyses for Figures 5(a-d) for both the cell lines; A one-way ANOVA followed by Tukey's multiple comparisons post hoc tests were performed. All the treatment points and significance were compared to untreated control. * $p < 0.05$; ** $p < 0.01$; and **** $p < 0.0001$, ns—not significant vs. untreated control. n = 9.

Figure 5(a): T98G cells

Tukey's multiple comparisons test	Mean Diff.	95.00% CI of diff.	Significant?	Summary	Adjusted P Value
Control vs. TMZ only	31.51	23.49 to 39.53	Yes	****	<0.0001
Control vs. 30 sec	53.13	45.11 to 61.15	Yes	****	<0.0001
Control vs. 60 sec	62.48	54.46 to 70.50	Yes	****	<0.0001
Control vs. 120 sec	72.98	64.96 to 81.00	Yes	****	<0.0001
Control vs. 1 day	61.96	53.94 to 69.98	Yes	****	<0.0001
Control vs. 2 days	62.48	54.46 to 70.49	Yes	****	<0.0001

Figure 5(b): A172 cells

Tukey's multiple comparisons test	Mean Diff.	95.00% CI of diff.	Significant?	Summary	Adjusted P Value
Control vs. TMZ only	39.39	27.11 to 51.67	Yes	****	<0.0001
Control vs. 30 sec	11.84	-0.4420 to 24.11	No	ns	0.0622
Control vs. 60 sec	30.42	18.14 to 42.69	Yes	****	<0.0001
Control vs. 120 sec	61.63	49.35 to 73.91	Yes	****	<0.0001
Control vs. 1 day	63.95	51.67 to 76.23	Yes	****	<0.0001
Control vs. 2 days	72.27	59.99 to 84.55	Yes	****	<0.0001

Figure 5(c): T98G cells

Tukey's multiple comparisons test	Mean Diff.	95.00% CI of diff.	Significant?	Summary	Adjusted P Value
Control vs. TMZ only	31.43	22.60 to 40.27	Yes	****	<0.0001
Control vs. 1 Day	40.65	31.81 to 49.48	Yes	****	<0.0001
Control vs. 2 Days	16.96	8.122 to 25.79	Yes	***	0.0006
Control vs. 3 Days	2.166	-6.670 to 11.00	No	ns	0.9227
TMZ only vs. 1 Day	9.217	0.3805 to 18.05	Yes	*	0.0401
TMZ only vs. 2 Days	-14.47	-23.31 to -5.637	Yes	**	0.0022
TMZ only vs. 3 Days	-29.27	-38.10 to -20.43	Yes	****	<0.0001
1 Day vs. 2 Days	-23.69	-32.53 to -14.85	Yes	****	<0.0001
1 Day vs. 3 Days	-38.48	-47.32 to -29.65	Yes	****	<0.0001
2 Days vs. 3 Days	-14.79	-23.63 to -5.957	Yes	**	0.0019

Figure 5(d): A172 cells

Tukey's multiple comparisons test	Mean Diff.	95.00% CI of diff.	Significant?	Summary	Adjusted P Value
Control vs. TMZ only	39.39	30.05 to 48.73	Yes	****	<0.0001
Control vs. 1 Day	62.92	53.58 to 72.26	Yes	****	<0.0001
Control vs. 2 Days	66.42	57.08 to 75.75	Yes	****	<0.0001
Control vs. 3 Days	77.33	68.00 to 86.67	Yes	****	<0.0001
TMZ only vs. 1 Day	23.53	14.19 to 32.87	Yes	****	<0.0001
TMZ only vs. 2 Days	27.03	17.69 to 36.36	Yes	****	<0.0001
TMZ only vs. 3 Days	37.94	28.61 to 47.28	Yes	****	<0.0001
1 Day vs. 2 Days	3.497	-5.840 to 12.83	No	ns	0.7343
1 Day vs. 3 Days	14.41	5.078 to 23.75	Yes	**	0.0034
2 Days vs. 3 Days	10.92	1.581 to 20.25	Yes	*	0.0211

Table S5. Statistical analyses for Figures 6(a-d) for both the cell lines; Comparison of treatments between all the groups and untreated controls. A one-way ANOVA followed by Tukey's multiple comparisons post hoc tests were performed. * $p < 0.05$; ** $p < 0.01$; and **** $p < 0.0001$, ns—not significant vs. untreated control. n = 9.

Figure 6(a): T98G cells

Tukey's multiple comparisons test	Mean Diff.	95.00% CI of diff.	Significant?	Summary	Adjusted P Value
Control vs. TMZ only	29.54	21.99 to 37.09	Yes	****	<0.0001
Control vs. 30 Sec	36.37	28.82 to 43.92	Yes	****	<0.0001
Control vs. 60 Sec	34.89	27.34 to 42.44	Yes	****	<0.0001
Control vs. 90 Sec	38.2	30.65 to 45.75	Yes	****	<0.0001
Control vs. 120 Sec	41.24	33.69 to 48.79	Yes	****	<0.0001

Figure 6(b): A172 cells

Tukey's multiple comparisons test	Mean Diff.	95.00% CI of diff.	Significant?	Summary	Adjusted P Value
Control vs. TMZ only	39.39	26.47 to 52.31	Yes	****	<0.0001
Control vs. 30 Sec	23.68	10.76 to 36.60	Yes	***	0.0005
Control vs. 60 Sec	29.44	16.52 to 42.36	Yes	****	<0.0001
Control vs. 90 Sec	38.03	25.12 to 50.95	Yes	****	<0.0001
Control vs. 120 Sec	45.06	32.14 to 57.97	Yes	****	<0.0001

Figure 6(c): T98G cells

Tukey's multiple comparisons test	Mean Diff.	95.00% CI of diff.	Significant?	Summary	Adjusted P Value
Control vs. TMZ only	26.45	23.10 to 29.79	Yes	****	<0.0001
Control vs. 1 Time CAP	33.21	29.86 to 36.55	Yes	****	<0.0001
Control vs. 2 Times CAP	38.22	34.87 to 41.57	Yes	****	<0.0001
Control vs. 3 Times CAP	44.08	40.73 to 47.43	Yes	****	<0.0001
TMZ only vs. 1 Time CAP	6.761	3.414 to 10.11	Yes	***	0.0004
TMZ only vs. 2 Times CAP	11.77	8.427 to 15.12	Yes	****	<0.0001
TMZ only vs. 3 Times CAP	17.63	14.28 to 20.98	Yes	****	<0.0001
1 Time CAP vs. 2 Times CAP	5.013	1.666 to 8.360	Yes	**	0.0042
1 Time CAP vs. 3 Times CAP	10.87	7.524 to 14.22	Yes	****	<0.0001
2 Times CAP vs. 3 Times CAP	5.858	2.511 to 9.205	Yes	**	0.0013

Figure 6(d): A172 cells

Tukey's multiple comparisons test	Mean Diff.	95.00% CI of diff.	Significant?	Summary	Adjusted P Value
Control vs. TMZ only	42.72	35.67 to 49.78	Yes	****	<0.0001
Control vs. 1 Time CAP	26.41	19.35 to 33.46	Yes	****	<0.0001
Control vs. 2 Times CAP	34.09	27.04 to 41.14	Yes	****	<0.0001
Control vs. 3 Times CAP	42.55	35.49 to 49.60	Yes	****	<0.0001
TMZ only vs. 1 Time CAP	-16.31	-23.37 to -9.261	Yes	***	0.0001
TMZ only vs. 2 Times CAP	-8.634	-15.69 to -1.581	Yes	*	0.016
TMZ only vs. 3 Times CAP	-0.1754	-7.229 to 6.878	No	ns	>0.9999
1 Time CAP vs. 2 Times CAP	7.681	0.6271 to 14.73	Yes	*	0.0317
1 Time CAP vs. 3 Times CAP	16.14	9.086 to 23.19	Yes	***	0.0002
2 Times CAP vs. 3 Times CAP	8.459	1.405 to 15.51	Yes	*	0.0181

Table S6. Statistical analyses for Figures 8(c,d) for both the cell lines; Comparison of treatments between all the groups. A one-way ANOVA followed by Tukey’s multiple comparisons post hoc tests were performed. All the treatment points and significance were compared to untreated control. * $p < 0.05$; ** $p < 0.01$; and **** $p < 0.0001$, ns—not significant vs. untreated control. n = 9.

Figure 8(c)

Tukey's multiple comparisons test	Mean Diff.	95.00% CI of diff.	Significant?	Summary	Adjusted P Value
Control vs. TMZ only	19.65	5.110 to 34.19	Yes	*	0.0108
Control vs. CAP only	-11.36	-25.90 to 3.185	No	ns	0.1342
Control vs. CAP+TMZ	-59.42	-73.96 to -44.87	Yes	****	<0.0001
TMZ only vs. CAP only	-31.01	-45.55 to -16.47	Yes	***	0.0006
TMZ only vs. CAP+TMZ	-79.07	-93.61 to -64.53	Yes	****	<0.0001
CAP only vs. CAP+TMZ	-48.06	-62.60 to -33.52	Yes	****	<0.0001

Figure 8(d)

Tukey's multiple comparisons test	Mean Diff.	95.00% CI of diff.	Significant?	Summary	Adjusted P Value
Control vs. TMZ only	-29.66	-37.41 to -21.90	Yes	****	<0.0001
Control vs. CAP only	-6.788	-14.54 to 0.9668	No	ns	0.0878
Control vs. CAP+TMZ	-69.71	-77.46 to -61.95	Yes	****	<0.0001
TMZ only vs. CAP only	22.87	15.11 to 30.62	Yes	****	<0.0001
TMZ only vs. CAP+TMZ	-40.05	-47.80 to -32.29	Yes	****	<0.0001
CAP only vs. CAP+TMZ	-62.92	-70.67 to -55.16	Yes	****	<0.0001
